# P09 Exploring prescribers’ reasons for prescribing piperacillin/tazobactam in Cwm Taf Morgannwg University Health Board

**DOI:** 10.1093/jacamr/dlae217.013

**Published:** 2025-01-23

**Authors:** Sian Price

**Affiliations:** Cwm Taf Morgannwg University Health Board, Abercynon, Wales

## Abstract

**Background:**

Using broad-spectrum antibiotics such as piperacillin/tazobactam (TZP) increases the risk of antibiotic-resistant infections and *Clostridioides difficile* infection.^1^ Appropriate antibiotic prescribing in Cwm Taf Morgannwg University Health Board (CTMUHB) is regarded as being in line with one or more of the following recommended prescribing principles (RPP):^2^ in accordance with CTMUHB antimicrobial guidelines;^3^ in accordance with culture and sensitivity results; or on the advice of a microbiologist. A snapshot audit in Princess of Wales Hospital in 2022 classed 9 out of 16 (56%) of TZP prescriptions as inappropriate.^4^

**Objectives:**

To explore and describe prescribers’ reasons behind prescribing TZP outside of the RPP in CTMUHB.

**Methods:**

A quantitative, cross-sectional study was conducted across the three acute sites in the Health Board, surveying all medical and non-medical prescribers (NMPs). An electronic (Microsoft Forms) self-completed, anonymous questionnaire with quantitative and qualitative questions was used to collect responses. Nominal (categorical) and ordinal (Likert scale) data were analysed and reported in terms of frequencies and percentages. Thematic analysis was used to examine the qualitative data.

**Results:**

A total of 92 prescribers (66 medical, 26 non-medical) were included in the data analysis. Of these, 96% (*n*=88) stated that they were aware of the CTMUHB antimicrobial guidelines and knew how to access them. Fifty-five percent of respondents (*n*=51) had prescribed TZP outside of the RPP (71% of medical prescribers and 15% of NMPs). The most common reason for this was ‘consultant/senior preference’ (59% of respondents). This reason was given by 25% of NMPs (1/4) compared with 62% (29/47) of medical prescribers.

**Conclusions:**

‘Prescribing etiquette’ and medical hierarchy in secondary care appears to be the biggest influence on prescribing^5^ with ‘consultant/senior preference’ being the most common reason given for prescribing outside of the RPP. This practice seems to impact medical prescribers more than NMPs. Further research in the form of focus groups or interviews with senior doctors is required to explore in more depth the reasons why they use TZP outside of the RPP.
